# Exploring the potential of psychedelics in brain injury recovery: a thematic analysis of participant narratives

**DOI:** 10.3389/fneur.2026.1880026

**Published:** 2026-09-16

**Authors:** Oliver Reed

**Affiliations:** Independent Researcher, Ashbourne, United Kingdom

**Keywords:** trauma, mental health, neurorehabilitation, psychedelics, traumatic brain injury (TBI)

## Abstract

Individuals with traumatic brain injury (TBI) frequently experience persistent cognitive difficulties, emotional dysregulation, and trauma-related symptoms, yet few interventions effectively address these interconnected challenges. This exploratory qualitative study examined how people with TBI perceived the effects of psychedelic use on cognition, mental health, and trauma recovery. Five adults with a history of TBI who had used psychedelics post-injury participated in semi-structured interviews. Data were analysed using reflexive thematic analysis. Three themes were identified: (1) Trauma Relief and Mood Stabilisation, describing reductions in anxiety, emotional reactivity, and trauma-related distress; (2) Perceived Post-Dose Cognitive ‘Lift’ particularly in memory, clarity, and coordination following macrodose experiences; and (3) Insight, Acceptance, and Self-Compassion, reflecting enhanced self-understanding, identity reconstruction, and gentler relationships with the self after injury. Participants reported that macrodose experiences produced the most noticeable effects, including perceived short-term improvements in memory, clarity, and coordination. Overall, participants described psychedelic experiences as associated with trauma relief and mood stabilisation, perceived cognitive changes, and greater insight, acceptance, and self-compassion following TBI, with potential implications for neurorehabilitation.

## Introduction

Traumatic brain injury (TBI) is a form of acquired brain injury (ABI) that occurs after birth and results from an external mechanical force causing damage to brain tissue and sustained neurological impairment (1). Common causes include falls, assaults, and traffic accidents, all of which can produce varying degrees of focal or diffuse injury (1). TBI is a highly heterogeneous condition: its presentation and prognosis depend on the nature, location, and severity of the impact, as well as secondary physiological and psychological consequences (2).

TBI frequently results in complications associated with frontotemporal dysfunction, leading to emotional and behavioural disturbances such as depression, anxiety, irritability, and aggression (1). Senathi-Raja et al. (3) found that long-term TBI survivors exhibited significantly poorer cognitive outcomes than those more recently injured, regardless of age. Neuropsychiatric disorders such as depression are also highly prevalent and can substantially hinder rehabilitation and quality of life; Singh et al. (4) reported that 56.3% of 827 participants met criteria for depression.

TBI is also associated with elevated risk of post-traumatic stress disorder (PTSD). A meta-analysis of civilian TBI cases reported a pooled PTSD prevalence of approximately 15.6% across injury severities, with minimal difference between mild and moderate/severe injuries (13.5% vs. 11.8%; (5)). Cohort studies indicate that TBI survivors show significantly higher rates of PTSD than patients with non-head trauma, reinforcing the need for trauma-informed rehabilitation pathways and integrated psychological support (6). Together, the cognitive, affective, and trauma-related burden of TBI highlights the need for approaches addressing both neurological and psychological recovery.

In recent years, a renewed scientific interest in classical psychedelics including psilocybin, lysergic acid diethylamide (LSD), N, N-dimethyltryptamine (DMT), and mescaline has positioned them as potential tools for both psychiatric and neurological recovery. These compounds act primarily as 5-HT₂A receptor agonists, modulating serotonin pathways that influence emotional regulation, perception, and learning (7). Neuroimaging studies demonstrate that psychedelics transiently alter default mode network activity and connectivity while increasing communication across previously segregated networks (8), changes proposed to contribute to enhanced cognitive flexibility and emotional openness.

At the cellular level, psychedelics appear to induce structural and functional neuroplasticity. Preclinical work has shown that compounds such as psilocybin and LSD promote synaptogenesis and dendritic spine growth via serotonergic and glutamatergic signalling pathways, enhancing the brain’s capacity for re-learning and adaptation (9, 10). LSD has been associated with increased expression of brain-derived neurotrophic factor (BDNF)—a biomarker of neuronal growth and resilience, indicating a potential molecular basis for its enduring cognitive and mood effects (10).

Clinically, psychedelics have demonstrated promising therapeutic outcomes. In a landmark open-label trial, Carhart-Harris et al. (8) demonstrated that psilocybin administered with psychological support produced rapid and sustained reductions in depressive symptoms in individuals with treatment-resistant depression. Follow-up research by Gukasyan et al. (11) extended these findings, showing that psilocybin produced significant antidepressant effects lasting up to 12 weeks with minimal adverse events. Beyond depression, controlled studies report benefits in anxiety (12, 13) and PTSD, particularly with MDMA-assisted therapy (14). Psilocybin has also been linked to enduring positive shifts in personality traits, such as increased openness and nature-relatedness, highlighting potential mechanisms for enhanced wellbeing and motivation (15).

Together, this body of evidence indicates that psychedelics exert their therapeutic effects through a dual process of biological and psychological plasticity. Biologically, serotonergic and neurotrophic modulation may enhance synaptic growth and network integration, processes that are crucial for restoring cognitive functions such as attention, memory, and executive control after brain injury. Psychologically, the transiently flexible brain state produced by psychedelics appears to facilitate emotional processing, trauma resolution, and adaptive reframing, factors that directly influence mental health recovery. For individuals with TBI who often experience persistent cognitive deficits, affective dysregulation, and trauma-related distress, these combined mechanisms may offer a novel pathway to rehabilitation. By potentially influencing neural plasticity alongside emotional processing, psychedelics may offer mechanisms relevant to new learning, mood regulation, and engagement with therapeutic and behavioural interventions.

Ly et al. (9) demonstrated that compounds such as LSD and DMT significantly increased dendritic spine growth and promoted synaptogenesis in rodent cortical neurons, leading to enhanced connectivity and structural plasticity. Potential relevance to brain injury has also been proposed through neuroinflammatory and neuroprotective pathways.

Allen et al. (16) summarised several preclinical studies showing that psilocybin, LSD, DMT, and 5-MeO-DMT reduced pro-inflammatory markers and limited neural tissue damage in animal models. For example, DMT was found to lower inflammation and reduce infarct size in a rat model of stroke, while psilocybin prevented toxin-induced increases in inflammatory markers in mice. Khan et al. (17) similarly identified neuroinflammation, neuroplasticity, hippocampal neurogenesis, and changes in brain complexity as candidate pathways through which psychedelics might influence neurological recovery.

Early human research has now begun to examine psychedelic interventions directly in brain-injured populations. Cherian et al. (18) investigated magnesium–ibogaine therapy in 30 Special Operations Forces veterans with predominantly mild TBI, reporting significant improvements in cognitive functioning, disability, PTSD, depression, and anxiety at one-month follow-up. Subsequent investigations of the MISTIC cohort identified structural and functional neuroimaging changes following treatment, including increased cortical thickness and subcortical volumes, reduced predicted brain age, and alterations in cerebral blood flow and functional connectivity (19). Longer-term follow-up indicated that improvements in disability, PTSD, depression, and anxiety remained evident at 12 months (20). Related findings have emerged from Special Operations Forces veterans receiving ibogaine and 5-MeO-DMT, with prospective improvements across mental-health, cognitive, and neurobehavioural outcomes (21). Blest-Hopley et al. (22) reported improvements in PTSD, depression, and anxiety alongside EEG changes in veterans with TBI histories following psilocybin retreats. Beyond macrodose interventions, a recent three-person case series reported neurological and psychological improvements following a six-week iboga microdosing protocol in individuals with TBI or hypoxic brain injury (23). Collectively, these studies provide an emerging human evidence base supporting further investigation in brain-injury populations.

Despite these promising signals, no qualitative studies have yet explored how individuals with TBI themselves perceive psychedelic use and its relationship to recovery. Understanding these lived experiences may help identify potential benefits, risks, and areas for future neurorehabilitation research.

Accordingly, the present study aims to explore the self-reported effects of psychedelics on cognitive functioning, mental health symptoms, and trauma recovery in individuals with TBI. By examining these subjective accounts through thematic analysis, this research seeks to generate preliminary insight into perceived neurocognitive and emotional changes and their potential relevance to neurorehabilitation.

## Methods

### Design

This qualitative study used a semi-structured interview design and reflexive thematic analysis (25) to explore how individuals with TBI experienced psychedelic use in relation to cognitive functioning, mental health, and trauma recovery.

The approach was interpretive and exploratory, aiming to identify common patterns of meaning while allowing space for individual nuance and lived experience.

### Participants

Five participants (*n* = 5) took part in the study, all of whom reported a history of TBI, including injuries sustained through events such as road traffic accidents and physical trauma. Although the original recruitment framework permitted inclusion of individuals with ABI more broadly, all participants recruited fell within the TBI category.

All participants reported using psychedelic substances following their injury, including psilocybin, LSD, and DMT. Variation was observed in both frequency and context of use, with participants reporting experiences ranging from one-off use to repeated sessions across recreational, self-directed therapeutic, and retreat settings.

Further details of participant characteristics, including age range, time since injury, injury characteristics, and patterns of psychedelic use, are presented in Table 1. Formal acute TBI severity classifications were not available for all participants. As injury histories were collected retrospectively through self-report, available clinical indicators of injury severity, including loss of consciousness or coma, intracranial injury, neurosurgical intervention, hospitalisation, and rehabilitation history, are reported descriptively rather than being used to retrospectively assign participants to formal severity categories.

**Table 1:** Participant demographic, injury, and psychedelic-use characteristics.

Participant ID	Age range	Time since injury	First Psychedelic experience after injury	Type of injury	Injury characteristics/severity indicators	Psychedelic use pattern/frequency
P1	20–30	8 years	6 years	TBI	Diffuse axonal injury; 2-week coma; 6-month hospitalisation	Multiple psychedelic experiences: frequency not systematically reported
P2	30–40	19 Years	2–3 Years	TBI	Childhood depressed skull fracture with LOC; subsequent skull fracture and intracranial haemorrhage; 10-day hospitalisation; post-injury seizures	Occasional/repeated use; LSD several times, psilocybin several times, including multiple synthetic psilocybin experiences
P3	40–50	15–20 Years	1–2 Years	TBI	2.5-day coma; initial wheelchair use; subsequent neurorehabilitation	Repeated psychedelic use; intermittent/irregular microdosing
P4	50–60	40 Years	40 Years	TBI	Intracranial haemorrhage and secondary stroke; burr-hole surgery; several days of life support; prolonged post-injury amnesia	Infrequent macrodose use, 1 year between initial macrodoses; 4–5 months of microdosing
P5	30–40	15 Years	5 Years	TBI	Major traumatic injury requiring resuscitation and hospitalisation; prolonged initial functional impairment	Repeated macrodose use; intermittent microdosing when undertaken

### Inclusion criteria

Eligible participants were required to be 18 years or older, have a documented history of ABI or TBI (traumatic or non-traumatic), and to have used psychedelic substances after the injury. Participants also needed sufficient English proficiency to understand the study information and provide informed consent. Individuals were excluded if they were under the age of 18, had only used psychedelics prior to their injury, reported no cognitive or psychological symptoms following the injury, or presented with communication or cognitive impairments severe enough to preclude meaningful participation in an interview. Demographic information was self-reported at interview, and all participants created a pseudonymous identification code (last three letters of surname plus last three digits of phone number) to preserve confidentiality across consent, transcription, and analysis materials.

### Recruitment

Participants were recruited through psychedelic community networks and online outreach between 2024 and 2025. Three participants were met personally within psychedelic community spaces and retreats where informal discussions about research interest occurred. One participant responded to a WhatsApp advertisement shared through the UK Psychedelic Society network, and another was recruited via peer referral from a previous participant who met inclusion criteria. Interested individuals received the Participant Information Sheet, Consent Form, and Debrief documents electronically prior to participation. Each participant confirmed voluntary consent and understanding of the withdrawal procedure, including their right to withdraw within 14 days of the interview. For appreciation for their time, participants received a £25 Amazon voucher upon completion.

### Data collection

Interviews were conducted online via secure video call (Zoom), lasted approximately 60 min, and were audio-recorded with permission. A semi-structured interview schedule (Appendix A) guided the conversation across five key domains: participants’ injury and recovery background (including the nature of the injury and ongoing symptoms); psychedelic use and context (substances used, dosage, intentions, and set/setting); perceived cognitive effects (attention, memory, processing speed, and daily functioning); mental-health and emotional changes (mood, anxiety, depression, and trauma-related experiences); and integration and reflection (meaning-making, quality of life, and self-concept). Prompts were used flexibly to encourage elaboration and depth while allowing participants to lead the discussion where relevant. Field notes were written immediately after each interview to capture contextual impressions and early analytic insights. All interviews were transcribed verbatim, anonymised, and stored on an encrypted drive.

### Researcher reflexivity

I approached the research with a pre-existing interest in, and broadly favourable orientation towards, the therapeutic potential of psychedelics, informed partly by personal experience of their perceived psychological benefits. Prior to data collection, I anticipated that participants might describe changes in cognition, mood, and trauma-related symptoms, reflecting the original research aims. I therefore remained aware that these expectations could influence the areas I attended to during interviews and subsequent interpretation of the data.

Changes in insight and self-understanding were not anticipated prior to data collection but emerged spontaneously during the first interview. This prompted me to add the question, “Have you noticed any changes in how you understand your brain injury or how it has affected you since using psychedelics?” to subsequent interviews. I recognised that explicitly introducing this topic may have contributed to its prominence within the final analysis.

I also became aware during interviewing that some participants appeared particularly motivated to communicate a positive narrative regarding psychedelics. This prompted reflection on whether some accounts may have emphasised or overstated perceived benefits, particularly given recruitment through psychedelic-engaged communities. Throughout the research process, I used reflexive notes and recorded reflections to consider these influences and to distinguish participants’ reported experiences from my own theoretical interpretations.

### Data analysis

Transcripts were analysed using reflexive thematic analysis following the six-phase framework outlined by Braun and Clarke (25) and Braun and Clarke (24). The process began with repeated reading of each transcript to ensure deep familiarisation with the data and to note early analytic impressions. Inductive, line-by-line coding was then undertaken manually, with attention given to segments describing cognitive changes, emotional shifts, trauma processing, and recovery-related reflections. Codes were subsequently collated into provisional clusters and iteratively refined into candidate themes, which were reviewed for internal coherence and distinctiveness in relation to the raw data. Once developed, themes were further defined and named to reflect participant’s own phrasing and the conceptual nuance of their accounts. Throughout the analysis, reflexive memo-writing supported interpretative decision-making and enhanced analytic rigour.

Given the exploratory nature of the study and the narrowly defined research question, the analysis prioritised depth of individual accounts and identification of preliminary patterns across participants rather than seeking statistical representativeness or formal data saturation. The final sample of five participants generated sufficiently detailed accounts to identify recurring patterns relevant to the study aims, while also retaining variation in individual experiences. However, data saturation was not formally assessed, and the themes should therefore be understood as exploratory rather than exhaustive representations of psychedelic experiences following brain injury.

### Ethics

The study was conducted independently and was not affiliated with a university or other institution through which formal research ethics committee review could be obtained. Consequently, the study did not receive independent institutional ethics approval. Ethical procedures were instead developed with reference to the British Psychological Society’s Code of Human Research Ethics. Participants received written study information before participation, provided informed consent, were informed of their right to withdraw within 14 days following interview, and were informed of procedures relating to confidentiality and data handling. Participation was voluntary, and the £25 voucher provided following interview was intended as compensation for the participant’s time.

## Results

Five participants (*n* = 5) with a history of TBI took part in the study. Participants were aged approximately 20–60, and time since injury ranged from approximately 8–40 years.

All participants reported using psychedelic substances following their injury, most commonly psilocybin and LSD, with some also reporting use of DMT. Frequency of use and patterns of psychedelic use varied across participants, ranging from intermittent or infrequent macrodose experiences to repeated psychedelic use and periods of microdosing. Contexts of use varied and included recreational use, self-directed therapeutic use, and retreat or guided settings.

No participants spontaneously reported adverse effects or persistent negative psychological reactions associated with psychedelic use during the interviews. However, adverse events were not systematically assessed as part of the interview schedule; therefore, this finding should not be interpreted as evidence of safety.

This variability reflects the exploratory and naturalistic nature of the sample. Participant characteristics are summarised in Table 1.

### Theme 1: trauma relief and mood stabilisation

Across participants with a history of TBI, accounts of psychedelic use frequently centred on changes in emotional regulation, particularly reductions in anxiety and trauma-related distress. These experiences were often described in the context of long-standing post-injury emotional difficulties.

Participants consistently described reductions in anxiety, emotional distress, or trauma-related symptoms following psychedelic use. These changes were often experienced as noticeable shifts in long-standing emotional patterns. For several participants, this involved a sense of calmness or emotional relief emerging in the hours or days after a psychedelic session.

One participant reflected on a marked change in anxiety levels following a psilocybin experience:

“After that first psilocybin session, I wasn’t waking up with the same anxiety—it felt like something had lifted.”—P4.

Another participant described improvements in post-traumatic stress symptoms, noting emotional clarity and reduced distress in the weeks that followed:

“It started my PTSD healing. In the weeks after, I feel calmer, clearer—like I’m not carrying the same weight.”—P5.

For some, these emotional changes were experienced as beneficial even when cognitive effects were less noticeable. One participant emphasised the mood-related improvements:

“I might not notice big ‘cognitive’ changes, but my mood is better after a session, and that changes how I cope.”—P2.

Participants with a history of anxiety or panic also described a shift in baseline emotional functioning:

“I’d had panic attacks and low mood for years. Psychedelics didn’t magically fix everything, but they shifted my emotional baseline.”—P1.

For one participant, the reduction in anxiety was described as immediate and significant:

“I’ve not had anxiety since I had that first psilocybin session… overnight, I was a different person.”—P4.

Others described greater day-to-day steadiness or less emotional reactivity:

“What changed was the emotional landscape. Less fear, more steadiness—that’s what stayed with me.”—P3.

Across all accounts, participants described psychedelic use as associated with calmer emotional states, reduced trauma-related burden, and improved mood stability.

Taken together, these accounts suggest that psychedelic experiences were not only associated with transient reductions in distress but may also have supported shifts in how participants related to long-standing emotional patterns following their injury.

### Theme 2: perceived post-dose cognitive ‘lift’

Participants with TBI frequently described a perceived shift in cognitive functioning following psychedelic use, often framed in relation to pre-existing post-injury difficulties such as memory impairment, reduced processing speed, or coordination challenges.

Participants described a temporary period of heightened cognitive clarity following psychedelic use. This “lift” was most often associated with macrodose experiences, with microdosing generally perceived as having minimal or no cognitive effect.

Several participants described sharper thinking, clearer recall, or improved coordination in the days and weeks following high-dose use. One participant highlighted striking improvements in short-term memory:

“After a macrodose, my short-term memory was noticeably better—and it lasted for months. Microdosing didn’t do that for me.”—P4.

Another participant described clearer cognitive functioning during boxing practice:

“In the weeks after mushrooms, I’m sharper—I can remember combinations in boxing and handle the cognitive load better.”—P5.

Others noted more general improvements in clarity:

“I get a kind of mental clarity in the days after. Not dramatic, but I think cleaner and make decisions easier.”—P2.

Participants also described everyday recall feeling easier or more fluid:

“It was like someone turned the memory dial up. Everyday things—names, tasks—came back faster.”—P4.

Coordination improvements were also noted:

“Right after a big dose, I’m quicker—timing, coordination, working memory—it’s like everything’s more in sync.”—P5.

Some described these effects as subtle but still noticeable in day-to-day functioning:

“I notice the clarity rather than a specific test score… day-to-day thinking just flows better.”—P2.

Importantly, these cognitive changes were often described relative to participants’ baseline difficulties following brain injury, suggesting that even subtle improvements were experienced as meaningful within the context of reduced cognitive capacity.

Several participants emphasised the temporary nature of the cognitive lift:

“For me it isn’t a constant cognitive boost—more of a window where things feel crisper, then it settles.”—P3.

Some cognitive effects lasted longer, particularly after macrodoses:

“My short-term memory was… noticeably different… maybe 4 or 5 months after a 4.5 g macrodose.”—P4.

And one participant described immediate improvements following high-dose use:

“It was the following week in the gym… timing, coordination… I could feel it instantly.”—P5.

However, participants varied in the duration and intensity of these effects, with some reporting short-lived periods of clarity lasting days, while others described more sustained changes extending over weeks or months.

Microdosing, by contrast, was widely reported as having little cognitive impact:

“Microdosing… didn’t seem to do anything at all.”—P4.

Overall, participants described psychedelic macrodoses as followed by a temporary period of cognitive clarity, especially in memory, processing, and coordination.

Taken together, these accounts suggest that psychedelic use was perceived as creating a temporary “window” of enhanced cognitive functioning, which participants often interpreted as a contrast to their usual post-injury cognitive limitations, rather than a permanent restoration of function.

### Theme 3: insight, acceptance, and self-compassion

Participants with TBI frequently described changes in self-perception, particularly in relation to how they understood their injury, their behaviour, and their sense of identity following psychedelic experiences.

Participants described gaining new insight into their injury, self-concept, or emotional patterns. Many spoke of developing greater self-acceptance or compassion, with psychedelics helping them relate differently to themselves and their lived experience of brain injury.

One participant described recognising behavioural and emotional responses in a new way:

“It opened a door in my mind. I could finally see how the injury shaped my behaviour—and stop beating myself up for it.”—P1.

Others described reconnecting with a more authentic sense of self:

“They helped me get back to who I am—that’s the best way I can put it.”—P2.

Participants also described shifts in perspective, particularly in relation to their injury:

“It was a paradigm shift. Not a symptom checklist—more a different way of relating to myself and my life.”—P3.

Self-compassion emerged as a prominent experience:

“The biggest change was accepting the injury. I could meet myself with more empathy instead of fighting it.”—P4.

Others described lasting emotional warmth toward themselves and those around them:

“I felt kinder—to myself and others. That self-compassion makes day-to-day life easier.”—P5.

Repeated psychedelic experiences were described as producing repeated openings or moments of clarity:

“Every time I take psychedelics it seems to open a door in my mind.”—P1.

These accounts were often situated within broader experiences of identity disruption following brain injury, where participants described difficulties reconciling their pre-injury and post-injury selves. Psychedelic experiences appeared to provide a context in which these discrepancies could be reflected upon with greater openness and reduced self-criticism.

Across participants, psychedelic use appeared to support reflection and emotional softening, allowing for increased acceptance and gentler self-understanding.

However, participants differed in how enduring these shifts were, with some describing lasting changes in self-perception, while others experienced these insights as temporary or requiring repeated experiences to maintain.

Taken together, these findings suggest that psychedelic experiences may have facilitated processes of psychological integration, enabling participants to reframe their relationship to their injury and develop a more compassionate and coherent sense of self.

### Summary

Participants’ experiences converged around three descriptive themes:

1. Trauma Relief and Mood Stabilisation
2. Perceived Post-Dose Cognitive ‘Lift’
3. Insight, Acceptance, and Self-Compassion

## Discussion

This study explored how individuals with TBI perceived the effects of psychedelic use on cognitive functioning, mental health, and trauma recovery. Three themes were identified: (1) Trauma Relief and Mood Stabilisation, (2) Perceived Post-Dose Cognitive ‘Lift’, and (3) Insight, Acceptance, and Self-Compassion. These findings provide preliminary qualitative insight into how participants perceived psychedelics as influencing neurocognitive and emotional recovery following brain injury.

### Trauma relief and mood stabilisation

One aim of the study concerned the potential impact of psychedelics on trauma-related distress and mental health difficulties commonly associated with brain injury. Participants described reductions in anxiety, emotional volatility, and trauma-related symptoms following psychedelic use. Feelings of calmness, emotional steadiness, and relief from long-held distress appeared across multiple accounts. These findings resonate with existing clinical research demonstrating the efficacy of psychedelics, particularly psilocybin and MDMA-assisted therapy, in reducing depression, anxiety, and PTSD symptoms (12–14).

Importantly, similar findings are now emerging in populations characterised by neurological and psychological trauma. Improvements in PTSD, depression, and anxiety have been reported following magnesium–ibogaine treatment in veterans with TBI (18), following psilocybin retreats in veterans reporting TBI histories (22), and following ibogaine and 5-MeO-DMT treatment in Special Operations Forces veterans (21). Notably, improvements in disability, PTSD, depression, and anxiety within the MISTIC cohort remained evident at 12-month follow-up (20), although intervening psychedelic use and other therapeutic interventions limit attribution of these longer-term outcomes specifically to ibogaine.

For individuals with TBI, emotional dysregulation, irritability, and trauma symptoms can significantly impair recovery and daily functioning. Participants’ experiences suggest that psychedelic use may be perceived as easing some of these emotional burdens, potentially supporting better engagement in rehabilitation, improved social interactions, and enhanced quality of life. The present qualitative findings complement this emerging quantitative literature by illustrating how participants themselves experienced these changes, including greater emotional steadiness, reduced fear and trauma-related burden, and an improved capacity to cope with life following injury.

Although these observations are based on retrospective accounts rather than controlled intervention, they highlight areas where psychedelic-assisted approaches may overlap with unmet clinical needs within neurorehabilitation.

### Perceived post-dose cognitive ‘lift’

A primary aim of this study was to explore whether psychedelics may support cognitive functioning after brain injury. Participants consistently described a period of improved clarity, memory, processing ability, and coordination following macrodoses, with these effects most noticeable in the immediate days or weeks after use. Importantly, participants framed these changes in relation to perceived post-injury cognitive difficulties, such as challenges with memory, coordination, and mental fatigue. These accounts align with preclinical research showing that psychedelics promote synaptogenesis, dendritic spine growth, and increased neural connectivity (9, 10).

Emerging human evidence provides further context: improvements in cognitive functioning have been reported following magnesium–ibogaine treatment in veterans with TBI (18), while subsequent structural and functional neuroimaging studies from the MISTIC cohort have identified measurable brain changes following treatment (19). Blest-Hopley et al. (22) similarly reported EEG changes following psilocybin retreats in veterans with TBI histories. Although these findings do not establish a mechanism for the subjective cognitive improvements reported here, they provide convergent clinical and neurobiological rationale for further investigation.

Although these cognitive effects were generally described as short-term, lasting from several days to a few months, participants described variability in both intensity and duration. The concentration of perceived cognitive changes within the post-dose period provides further rationale for investigating whether these effects could have relevance to neurorehabilitation. The comparatively limited effects attributed to microdosing in the present sample are also noteworthy considering recent case-series evidence. Tabaac et al. (23) reported neurological and psychological improvements following a six-week integrative iboga microdosing protocol in three individuals with TBI or hypoxic brain injury. Differences in compound, dosing, therapeutic support, and study design may contribute to these contrasting observations and warrant controlled investigation.

### Insight, acceptance, and self-compassion

Insight, acceptance, and self-compassion was not part of the study’s initial aims but emerged strongly from participant narratives. Many described psychedelic experiences as helping them understand how their injury had affected them, recognise emotional and behavioural patterns, and reconnect with a sense of identity or inner clarity that had felt diminished since the injury.

Identity disturbance is a well-documented consequence of brain injury, with survivors often reporting feelings of fragmentation, loss of self, or disconnection from who they were before the injury. Participants’ accounts suggest that psychedelic experiences may facilitate processes of psychological integration, allowing individuals to reflect on their injury with greater gentleness, acceptance, and contextual understanding. Increased self-compassion and reduced self-blame were common descriptions, and several participants emphasised feeling more connected to themselves or more aligned with a sense of personal authenticity.

These findings parallel broader psychedelic research showing increases in psychological flexibility, emotional openness, and self-related insight. While these effects are consistent with findings in non-brain-injured populations, their manifestation within individuals with traumatic brain injury suggests potential relevance to identity reconstruction and emotional recovery following neurological trauma.

### Integrating the findings: potential implications for TBI recovery and neurorehabilitation

#### Trauma relief and mood stabilisation

- Potential for reducing anxiety, fear, and trauma-related symptoms following TBI.
- Potential to support engagement in rehabilitation and daily functioning.

#### Perceived post-dose cognitive ‘lift’

- Potential relevance to cognitive domains commonly impaired following TBI.
- Potential for improving aspects of cognitive functioning following injury.
- Rationale for investigating whether these effects could support neurorehabilitation.

#### Insight, acceptance, and self-compassion

- Greater acceptance of injury.
- Renewed connection to self.
- Reduced self-blame and potential relevance to psychological adjustment following TBI.

Together, these domains highlight several potential areas of relevance to TBI recovery and neurorehabilitation. Trauma relief and mood stabilisation may alleviate persistent emotional and trauma-related difficulties following injury; perceived improvements in cognitive functioning may have relevance to ongoing cognitive difficulties; and greater insight, acceptance, and self-compassion may support psychological adjustment to life after TBI.

### Safety and clinical considerations

Although no participants spontaneously reported adverse effects or persistent negative psychological reactions, safety was not systematically assessed in this study. This is particularly important in TBI populations, where neurological and psychiatric vulnerability may alter the risk profile of psychedelic use. Potential concerns include acute psychological distress or destabilisation, interactions with existing medications, and possible neurological risks in individuals with seizure susceptibility.

### Limitations

The small, self-selected sample limits generalisability. Although the original recruitment framework permitted inclusion of ABI more broadly, the sample consisted exclusively of individuals with TBI, limiting generalisability to non-traumatic ABI populations. Recruitment primarily through psychedelic community spaces, retreats, networks, and participant referral also introduces selection bias, as individuals engaged with these communities or willing to discuss psychedelic use may have been more likely to hold favourable attitudes or interpret their experiences as beneficial.

Participants used a range of psychedelic substances in uncontrolled, naturalistic settings, limiting attribution of experiences to specific compounds, doses, or contexts. Data were retrospective and reliant on self-report, introducing recall bias, and no objective cognitive measures were collected. Formal psychiatric diagnoses and comorbidities were not systematically assessed, limiting assessment of their potential influence on participants’ perceived responses.

Finally, the study was conducted independently without institutional research ethics committee review. Although procedures were informed by BPS ethical guidance and included written informed consent, confidentiality safeguards, voluntary participation, and withdrawal procedures, these are not equivalent to independent ethical oversight. This should be considered when interpreting the findings and highlights the importance of formal institutional ethical oversight in future research with this population.

### Future research

Future work should include controlled studies examining the effects of psychedelics on trauma-related distress and mood, cognitive functioning, and psychological adjustment in TBI populations. Mixed methods design with pre- and post-session cognitive testing would help determine whether subjective reports align with measurable improvements. Future studies should examine whether perceived cognitive “lifts” reported by participants correspond to objective changes in domains commonly affected by TBI, such as memory, attention, and processing speed. Longitudinal research should explore how insight, identity reconstruction, and self-compassion evolve over time after psychedelic use. Such work may be especially relevant given the well-documented challenges of identity disruption following brain injury. Given the preliminary nature of microdosing accounts in this sample, controlled microdosing trials may also clarify its potential role in ongoing cognitive support. Finally, research should investigate the potential integration of psychedelics within structured neurorehabilitation, exploring how trauma relief and mood stabilisation, perceived improvements in cognitive functioning, and greater insight, acceptance, and self-compassion may contribute to neurorehabilitation outcomes.

## Conclusion

Participants’ accounts suggest that psychedelic use was associated with trauma relief and mood stabilisation, a perceived post-dose cognitive ‘lift’, and greater insight, acceptance, and self-compassion following TBI. While these experiences are consistent with broader psychedelic research, their expression within a TBI population highlights a potentially important area for further investigation. These findings are preliminary but, considered alongside a rapidly developing clinical and neurobiological evidence base in TBI populations, highlight several potential avenues through which psychedelics could be investigated in relation to persistent post-injury difficulties. The present qualitative findings contribute lived-experience perspectives to this emerging literature and may also have implications for the future integration of psychedelic approaches within neurorehabilitation.

## Data Availability

The original contributions presented in the study are included in the article/Supplementary material, further inquiries can be directed to the corresponding author.
