# Supplementary material for: Exploring the potential of psychedelics in brain injury recovery: a thematic analysis of participant narratives
Source: Front Neurol. 2026 Sep 16;17:1880026. doi: 10.3389/fneur.2026.1880026 (PMC13624134; doi:10.3389/fneur.2026.1880026)
Supplement: Supplementary file 1 [file Supplementary_file_1.docx]

Appendices

Appendix A

Interview Questions

Intro & Context

1.	Could you tell me a little about your brain injury and your recovery journey so far?

2.	Could you tell me about the main symptoms or challenges you’ve experienced since your brain injury?

3.	When did you first start using psychedelics after your injury, and what prompted that decision?

Use & Experience

4.	Which psychedelics have you used, and in what context (e.g., microdosing, full experience, therapeutic, recreational)?

5.	What were your intentions, if any, when using psychedelics after your brain injury?

Cognition

6.	Have you noticed any changes in your memory, attention, or thinking since using psychedelics?

7.	Have you experienced any improvements or challenges in day-to-day functioning that you associate with psychedelic use?

Mental Health

8.	How has using psychedelics affected your mood or emotional wellbeing?

9.	Have you noticed any changes in anxiety, depression, or how you handle stress?

Integration & Reflection

10.	Looking back, how do you feel psychedelics have influenced your overall recovery or quality of life since your injury?

11.	Have you noticed any changes in how you understand your brain injury, or how its affected you since using psychedelics?

12.	Is there anything else about your experience that you feel is important to share, especially regarding your healing or recovery journey?

Appendix B

| **Theme** | **Participant** | **Verbatim Quote** |
| --- | --- | --- |
| Trauma Relief and Mood Stabilisation | P4 | “After that first psilocybin session, I wasn’t waking up with the same anxiety—it felt like something had lifted.” |
| Trauma Relief and Mood Stabilisation | P5 | “It started my PTSD healing. In the weeks after, I feel calmer, clearer—like I’m not carrying the same weight.” |
| Trauma Relief and Mood Stabilisation | P2 | “I might not notice big ‘cognitive’ changes, but my mood is better after a session, and that changes how I cope.” |
| Trauma Relief and Mood Stabilisation | P1 | “I’d had panic attacks and low mood for years. Psychedelics didn’t magically fix everything, but they shifted my emotional baseline.” |
| Trauma Relief and Mood Stabilisation | P3 | “What changed was the emotional landscape. Less fear, more steadiness—that’s what stayed with me.” |
| Perceived Post-Dose Cognitive ‘Lift’ | P4 | “After a macrodose, my short-term memory was noticeably better—and it lasted for months.” |
| Perceived Post-Dose Cognitive ‘Lift’ | P5 | “In the weeks after mushrooms, I’m sharper—I can remember combinations in boxing…” |
| Perceived Post-Dose Cognitive ‘Lift’ | P2 | “I get a kind of mental clarity in the days after.” |
| Perceived Post-Dose Cognitive ‘Lift’ | P4 | “It was like someone turned the memory dial up.” |
| Perceived Post-Dose Cognitive ‘Lift’ | P5 | “Timing, coordination, working memory—it’s like everything’s more in sync.” |
| Perceived Post-Dose Cognitive ‘Lift’ | P3 | “More of a window where things feel crisper, then it settles.” |
| Perceived Post-Dose Cognitive ‘Lift’ | P1 | “I notice the clarity rather than a test score… thinking flows better.” |
| Insight, Acceptance, and Self-Compassion | P1 | “It opened a door in my mind. I could finally see how the injury shaped my behaviour…” |
| Insight, Acceptance, and Self-Compassion | P2 | “They helped me get back to who I am…” |
| Insight, Acceptance, and Self-Compassion | P3 | “It was a paradigm shift… a different way of relating to myself and my life.” |
| Insight, Acceptance, and Self-Compassion | P4 | “The biggest change was accepting the injury… meeting myself with empathy.” |
| Insight, Acceptance, and Self-Compassion | P5 | “I felt kinder—to myself and others. That self-compassion makes day-to-day life easier.” |
